# Applications of Antioxidant Nanoparticles

**DOI:** 10.3390/antiox13091096

**Published:** 2024-09-10

**Authors:** Rita Cortesi, Maddalena Sguizzato

**Affiliations:** Department of Chemical, Pharmaceutical and Agricultural Sciences (DoCPAS), University of Ferrara, 44100 Ferrara, Italy; maddalena.sguizzato@unife.it

The regulation of numerous physiological processes is strictly dependent on the production of reactive oxygen species (ROS). Nonetheless, their overproduction could generate oxidative stress, responsible for the onset of different diseases [1]. Hence, a useful treatment approach for oxidative stress is the delivery of antioxidants. Generally, neutralization of ROS, chelating effect and enzyme inhibition are the three main mechanisms of action exerted by antioxidant molecules to inhibit the effect of ROS and the generation of oxidative stress.

In recent decades, the application of nanoparticles has shown a considerable deal of interest in the fields of pharmaceutics, cosmetics and food [2,3,4]. For instance, lipid nanoparticles, due to the “green” characteristics related to their biodegradation and non-toxicity, have received significant attention as a carrier for antioxidant molecules [5]. Over the past few decades, considerable achievements have been made in the development of antioxidant nanotherapies, exhibiting multiple advantages.

This Special Issue of *Antioxidants*, entitled “Applications of Antioxidant Nanoparticles”, includes thirteen research articles and two reviews, describing recent advances in the design, production, characterization, use, and effect of antioxidant nanoparticles, including polymeric or lipid-based nanoparticles, ROS-scavenging inorganic nanoparticles, organic nanoparticles with intrinsic antioxidant activity and drug-loaded antioxidant nanoparticles. In addition, the use of antioxidant nanoparticles in various fields, such the biomedical, pharmaceutical, cosmetic and food industries, and their administration through different routes have been considered.

Concerning the different routes of administration, the review of Qi and colleagues (contribution 14) focuses on the involvement of different types and delivery of antioxidants in dental procedures. Particularly the study of their mechanisms of action in the treatment of oxidative stress induced by dental restorations is also taken into consideration.

On the other hand, the review of Subroto and colleagues provides information on the current research on solid lipid nanoparticles (SLNs) for the encapsulation and delivery of active and antioxidant compounds. Various synthesis methods and application fields of SLNs were considered. It was underlined that fabrication design, which includes the selection of lipid matrices and surfactants and their preparation methods, determines the characteristics of SLNs. Therefore, an appropriate fabrication design can produce SLNs with stable active compounds and antioxidants that become suitable encapsulation systems for various applications or uses (contribution 15).

With regard to the research papers, they can be divided in some groups on the basis of the type of delivery system proposed, namely nanoparticles (contributions 1, 5, 6, 7, 8, 11 and 12), nanocomposites and nanoclusters (contributions 3 and 10), SEDDS and nanoemulsions (contributions 2, 4 and 9) and vesicles (contribution 13).

Among the nanoparticle group, Bellini et al. demonstrated that poly (lipoic acid)-based nanoparticles stabilized by Pluronic are able to effectively antagonize the formation of radicals and ROS. In addition, these nanoparticles inhibited the overproduction of ROS in tumor cells, human macrophages and rat ventricular myocytes. In addition their capture by macrophages and cardiomyocytes induce a better effect as compared to tumor cells and non-phagocytic leukocytes. Notably, these nanoparticles decreased the mitochondrial ROS generation induced by simulated ischemia/reperfusion injury in isolated cardiomyocytes, hence appearing to be an effective and cardiomyocyte-selective drug to protect against damages induced by post-ischemic reperfusion (contribution 1).

Another study, conducted by the group of Mawed and colleagues, investigated the ultrastructure changes in the ovary in response to exposure to zinc oxide nanoparticles and the effect during egg maturation and ovulation on the fertility of female zebrafish. They found that zinc oxide nanoparticles could induce cytotoxicity in the maturing oocyte by activating autophagy and apoptosis in a caspase-dependent manner and could induce oxidative stress by generating ROS (contribution 5).

The research by Tellechea et al. investigated the characteristics of a recombinant protein from the legume cowpea (*Vigna unguiculata*) immobilized on gold nanoparticles. Notably, the study, conducted as a function of nanoparticle surface chemistry and biofunctionalization methods, evidenced the importance of the interaction between the biomolecule and the gold nanoparticles for the functionality of the hybrid; hence, this strategy can be useful in the development of electrochemical biosensors in biomedical applications (contribution 6).

Otherwise, Kim and colleagues studied the use of α-bisabolol as a natural bioactive compound in bioproducts to minimize oxidative stress. In order to bypass its water-insolubility, α-bisabolol-loaded polyglyceryl-4 caprate nanoparticles were proposed, obtaining an improvement in long-term stability and antioxidant activity, and an outstanding antibacterial effect that could allow for the use of these nanoparticles in treating diseases related to various oxidative stresses as well as in many fields, such as the pharmaceuticals, cosmetics, and food industries (contribution 7).

The research by Ivanova’s group focused on the use of an environmentally friendly and cost-effective self-assembly nanoencapsulation technology based on zein to obtain multifunctional essential oil-loaded nanocapsules with antioxidant and bactericidal activity toward *Cutibacterium acnes*. The obtained results demonstrated that once incorporated into creams, the produced nanocapsules led to complete inhibition of *C. acnes* and demonstrated the capacity to scavenge ROS, thus preventing damage to human skin cells (contribution 8).

The study by De Luca and colleagues considered the phytochemical resveratrol loaded onto positive charged polymeric nanoparticles within an in situ gelled matrix to prolong the residence time of the active into the cornea, decrease the administration frequency and enhance the therapeutic effect. The obtained eye drop formulation could be employed to bypass the rapid clearance typical of solutions in the treatment of a number of inflammation- and oxidative stress-related diseases (contribution 11).

The last manuscript belonging to the nanoparticle group is that of Thirupathi and colleagues. The authors evaluated on the palatal wounds of Wistar rats the cytotoxic effect and the activity of gold nanoparticles reduced by the green synthesis method. They found that topical treatment with these nanoparticles potentially remitted the palatal wounds and the mucosal lesions of mouth, preventing inflammation or infections and facilitating the healing process (contribution 12).

The second group of delivery systems including nanocomposites and nanocluster includes the works of Alam et al. (contribution 3) and Ungor and colleagues (contribution 10). The former considers a novel copper–zinc–manganese trimetal oxide nanocomposite synthesized using the co-precipitation method, which was then characterized and tested in vitro, showing potent activity against *Escherichia coli* and a strong antioxidant effect where scavenging activity was observed. In contrast, the latter studied the improvement in antioxidant activity of a simple synthesis method for the preparation of Vitamin B1-stabilized few-atomic gold nanoclusters. The obtained nanostructure carries gold in the zero oxidation state, while Vitamin B1 is able to stabilize the metal core by coordinating pyrimidine-N. In this way the gold nanoclusters is able to enhance its antioxidant property as compared to pure Vitamin B1.

The third group includes SEDDS and nanoemulsions. Notably, self-emulsifying drug delivery systems (SEDDSs) are isotropic mixtures of oils, surfactants, solvents and co-solvents/surfactants, useful for improving the oral absorption of lipophilic drug compounds. Afifi and colleagues loaded β-Sitosterol glucoside on SEDDSs in order to ameliorate insulin resistance, protect against oxidative stress and restore pancreatic β-cell secretory function. The obtained results suggest the possible employment of these systems in diabetes progression (contribution 2).

Concerning nanoemulsions, the study of Das and colleagues investigated a nanosized food-grade quercetin-loaded nanoemulsion for its neuroprotective and cytotoxicity effects against *Caenorhabditis elegans* strains and human cancer cells, respectively. In vivo results showed that the quercetin-nanoemulsion potentially reduced the α-Syn aggregation, increased mitochondrial and fat content, and improved the lifespan in transgenic *C. elegans* strain. Moreover, it significantly down-regulates the ROS levels in wild-type *C. elegans strain*, therefore showing improved solubility, targeting and neuroprotective effects against the parkinsonian-induced model (contribution 4).

The study by Thuy et al. took into consideration the development of a mitochondria-targeting emulsion, using dequalinium and α-tocopherol succinate for cancer treatment. The authors found that this nanoemulsion effectively inhibited spheroid growth in the 3D model, as well as prevented the growth of tumor cells grafted onto zebrafish larvae, and showed promising potential for mitochondria-targeting and cancer treatment (contribution 9).

Lastly, another type of formulation is typified by vesicles, which are described in the study of Sguizzato et al. considering bilosystems to improve the water solubility of budesonide. The obtained systems showed good encapsulation efficiency and dimensional stability. Moreover, immunofluorescence experiments evidenced that bile acid-based vesicles containing the drug effectively decrease the inflammation induced by glucose oxidase stimuli and counteract intestinal cells ox-inflammatory damage (contribution 13).

In summary, with this Special Issue, we have tried to uncover the possible applications of different types of nanoparticles, or more precisely, “nanosystems”, in different fields, including the biomedical, pharmaceutical, cosmetic, as well as nutraceutical or food industries. The involvement of oxidative stress in different diseases, ranging from cutaneous damages to systemic pathologies and cancer, clearly suggests that the use of antioxidant nanosystems with targeted and controlled release could enhance the effect of common treatments. Indeed, the results discussed in this Special Issue unveil the multiple modes of action of a potential antioxidant activity performed by some molecules when delivered in different technological systems and support the ongoing research for a better understanding of optimal ways to exploit the activities of antioxidant molecules in everyday life. The correlation between oxidative stress and physiological processes or disease progression is still an appealing topic to be investigated, and the application of antioxidant nanoparticles has proven to be a promising strategy to adopt in particular when endogenous defenses are lacking.
